# Increased mutation rates and diversity are dominant features of *Geobacter* multiheme cytochromes

**DOI:** 10.1128/mbio.03394-25

**Published:** 2026-02-17

**Authors:** Ruth Starwalt-Lee, Jeffrey A. Gralnick, Daniel R. Bond

**Affiliations:** 1 BioTechnology Institute and Department of Plant and Microbial Biology, University of Minnesota 25793, St. Paul, Minnesota, USA; The University of Oklahoma, Norman, Oklahoma, USA

**Keywords:** electron transfer, *Geobacter*, multiheme cytochromes, bioinformatics

## Abstract

Multiheme cytochromes (MHCs) are the central catalysts of extracellular electron transfer and are uniquely abundant in the genomes of model Fe(III)-reducing organisms like *Geobacter sulfurreducens*. While specific functions for some multiheme cytochromes are known, the complex repertoire present in any genome makes annotation and prediction of electron transfer circuitry challenging. Here, we reveal patterns of conservation and rates of evolutionary change among multiheme cytochromes that help explain these difficulties. Using the Ppc and OmcS cytochromes as examples, we show that sequence-based methods of determining protein homology can be inadequate for distinguishing between cytochromes known to have differing functions. Importantly, using mutation rate analysis, we find that multiheme cytochromes in *Geobacter* and *Shewanella* exhibit increased mutation rates, which may lead to inaccurate homolog identification even between closely related organisms. Finally, an analysis of multiheme cytochrome diversity reveals that each *Geobacter* genome contains a high proportion of cytochromes that are unique to that individual species, suggesting a high rate of horizontal acquisition and gene loss. These increased mutational and genetic exchange rates will need to be properly accounted for in annotation tools before we can accurately ascribe function and catalog the complex repertoire of cytochromes essential to extracellular electron transfer.

**IMPORTANCE:**

Dissimilatory metal-reducing bacteria are found worldwide and encode diverse multiheme cytochromes with properties suitable for applications in bioremediation, bioenergy, and bioelectronics. We find that multiheme cytochromes involved in extracellular electron transfer show poor conservation, with significantly higher mutation rates than other elements of the proteome. This previously undescribed characteristic will limit the efficacy of standard methods of homolog annotation and database mining currently used to identify specific multiheme cytochromes. Our findings also suggest that a vast pool of undiscovered multiheme cytochromes are constantly being acquired and exchanged.

## INTRODUCTION

The discovery of extracellular electron transfer represented a significant advancement in our understanding of bacterial respiratory strategies and uncovered a natural resource for engineering the interface between biology and electricity (1–4). In gram-negative bacteria, electrons are transferred from cytoplasmic metabolism through a sequence of multiheme cytochromes (MHCs) located in the cytoplasmic membrane, periplasm, outer membrane, and extracellular space to insoluble electron acceptors such as iron or manganese oxides (1). These cytochromes also allow organisms to transfer electrons to partner organisms such as methanogens or to artificial electron acceptors like poised electrodes (1, 5–7). This ability to interact with electrodes is the basis of energy-capturing microbial fuel cells, while wire-forming polymeric nanowire cytochromes are being investigated for applications in bioelectronics (8, 9). Despite their environmental and technological importance, unraveling electron transfer in models such as *Geobacter sulfurreducens* and predicting function in other uncultivated species is slowed by the complex complement of multiheme cytochromes present in each genome.

Complicating genetic and functional analysis is the fact that many cytochromes appear to be the result of duplication events. *G. sulfurreducens* encodes several classes of cytochromes that contain up to six paralogs in the same genome. An instance common to *Geobacter* and its relatives is the existence of between 1 and 6 members of the Ppc family of triheme cytochromes, which are abundant periplasmic proteins needed to transfer electrons from cytoplasmic membrane proteins to cytochrome conduits in the outer membrane (10, 11). Due to the fact that at least one of five Ppc genes (PpcA–E) are needed for extracellular electron transfer to metals by *G. sulfurreducens* and that they are present in nearly every Desulfuromonadales genome, they are among the most common proteins linked to the study of electron transfer function in Desulfuromonadales (10).

Each Ppc is differentially regulated, showing unique redox potentials and binding interactions with other cytochromes (12–15). Thus, correct identification of the closest relative of each well-characterized *G. sulfurreducens* Ppc homolog in other *Geobacter* genomes is essential when mapping this functional information onto other species. However, as we describe below, such identification is difficult, if not impossible.

Similarly, the conductive-wire-forming hexaheme OmcS has three other homologs in the *G. sulfurreducens* genome. Of these, only OmcS is known to form a conductive extracellular nanowire (15, 16). In organisms related to *Geobacter*, identifying true OmcS cytochromes, indicating a possible nanowire, is also challenging and prone to mistaken functional predictions. Compared to respiratory enzymes such as nitrate reductase that act as reliable markers of metabolic function during metagenomic studies, unreliable cytochrome annotation creates the risk that BLAST and Hidden Markov Model (HMM)-based pipelines will generate excessive errors and generate incorrect functional predictions in metal-reducing bacteria (17).

This work analyzed a set of curated genomes to ask if (i) we can reliably assign function or identify true orthologs of multiheme cytochromes across genera and (ii) if poor sequence conservation is a widespread feature of cytochromes. Sequence-based homology is typically used for annotation and assignment of specific physiological processes across phylogenetically diverse populations. However, in test cases using periplasmic and nanowire cytochromes of closely related organisms, this did not always hold true and only showed promise among closely related species. As high-quality annotation is essential for data-mining genome repositories, it is critical that we understand the origins of this poor conservation before using protein prediction tools.

Our analysis finds evidence that increased mutation rates are a general feature of cytochromes in the model genera *Geobacter* and *Shewanella*. Further, this survey of cytochrome diversity in *Geobacter* and *Shewanella* revealed hundreds of multiheme cytochromes that were each unique to a particular genome, lacking homologs in other strains. The existence of a large cytochrome pangenome, evolving at rates that exceed those of standard respiratory proteins, implies an enormous sequence space is being explored by cytochromes. This diversity poses unique opportunities for environmental and bioelectronic applications but will require new bioinformatic approaches to properly categorize.

## RESULTS

### Can we distinguish true PpcA from periplasmic triheme cytochrome homologs across *Geobacter* species?

**Fig 1:**
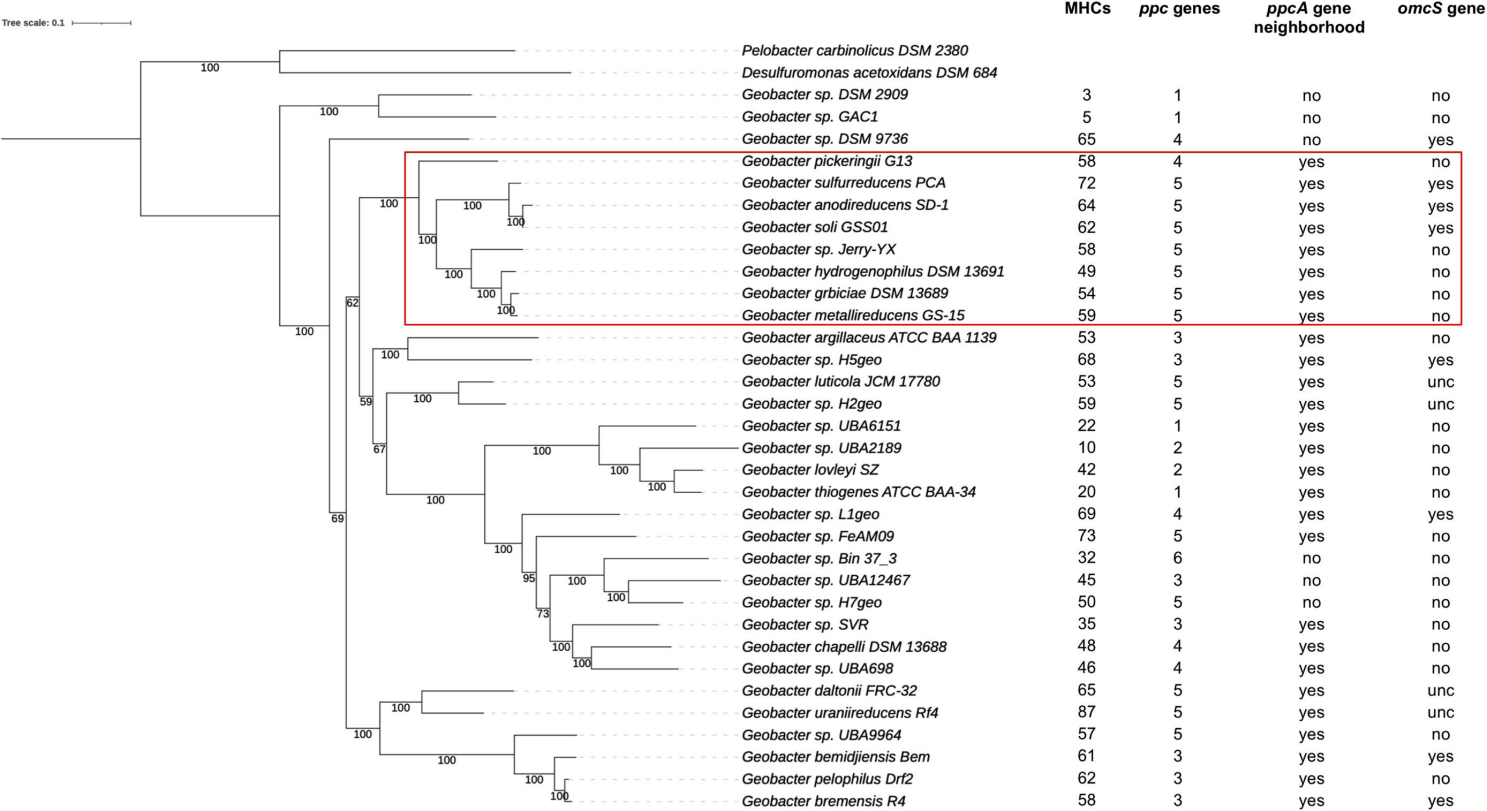
Maximum likelihood tree of *Geobacter* genomes used in this study. The box designates genomes in the “*G. sulfurreducens”* clade that retain the *Geobacter* genus in most current renaming schemes. Columns list the number of multiheme cytochromes (MHCs), putative periplasmic triheme *ppc* or hexaheme *omcS* genes, and whether a conserved PpcA gene neighborhood was found that allowed identification of putative PpcA orthologs. The tree branches are labeled with bootstrap values. Data for the *Desulfuromonas* outgroup are omitted from the analysis as only closely related *Geobacter* strains were used for this work.

Nearly every *Geobacter* genome encodes multiple triheme periplasmic cytochromes, with the largest family known by the gene designation *ppc*. In a curated set of 33 high-quality *Geobacter* genomes (see Materials and Methods), genomes encode an average of 4 *ppc* genes, with a range of 1–6 (Fig. 1). In *G. sulfurreducen*s, PpcA is the best characterized and most abundant in the periplasm (10, 18). When annotating a genome encoding multiple Ppcs, identifying the true PpcA ortholog is a challenge.

**Fig 2:**
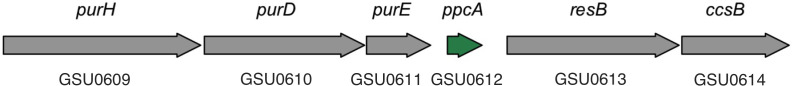
Conservation of synteny allowing identification of putative PpcA genes. The conserved *G. sulfurreducens ppcA* gene neighborhood.

For example, two triheme Ppc cytochromes in *G. metallireducens* have higher identity to *G. sulfurreducens* PpcA than to any other homolog in the *G. sulfurreducens* genome. Because these organisms are closely related, we can use conserved gene synteny around *G. sulfurreducens ppcA* to identify a likely true ortholog, allowing the remaining highly similar gene to be designated PpcB in *G. metallireducens* based on the next best hit (Fig. 2). However, such synteny information is rarely utilized in automated bidirectional best-hit algorithms, and as PpcA is not expressed as part of a larger operon, there is little reason for synteny to be maintained. Normally, when annotation software encounters two paralogs with higher similarity to PpcA than any other protein, it is treated as a duplication. In the case of *G. metallireducens,* PpcA and PpcB are simply labeled “PpcA_1” and “PpcA_2,” respectively. This obscures the protein’s evolutionary history and function. The error is amplified if *G. metallireducens* then acts as a reference sequence. If PpcA_2 (actually PpcB) has a best hit to a protein, the new gene will be named *ppcA*. Using a best-hit approach produces a worst-case outcome, as annotations rapidly become arbitrary.

To see how Ppc homologs challenge annotation algorithms, we tested the ability of standard bioinformatic approaches to identify PpcAs using both a genome annotation pipeline and a homolog clustering pipeline. First, Prokka was used to re-annotate a selection of high-quality *Geobacter* genomes using our manually annotated *G. sulfurreducens* PCA genome as the reference (19). In this workflow, Prokka identified all putative coding sequences and then annotated them based on their bidirectional best hit to the reference *G. sulfurreducens* genome.

Of 121 *ppc* annotations found across 33 genomes, 64 were annotated as *ppcA*. In 21 cases, between 2 and 4 genes were designated *ppcA,* while only nine genomes contained a single cytochrome annotated as *ppcA*. As expected, when we manually inspected genomes, most genes named *ppcA* did not share synteny with *G. sulfurreducens* PpcA, indicating they were not true orthologs (Fig. 2). The sequences of the Ppc cytochromes were in similarity ranges ~35%–100% (with BLOSUM62) that prevented clear bidirectional best hits to be resolved, causing them to be grouped together and given the same name.

These annotated *Geobacter* genomes were also analyzed with a bioinformatic pipeline (get_homologs), which creates homolog clusters from all-vs-all genome BLAST data using the Ortho Markov CLuster (OMCL) algorithm (20, 21). OMCL is not limited to pairwise comparisons. Because it considers an overall similarity network, it tends to be more sensitive in identifying orthologous genes in the presence of paralogs. This clustering is also better suited to variations in evolutionary distances between genes, making it more effective across a range of species divergence (21).

Using the OMCL algorithm, the majority of triheme cytochromes (58 of 121) still grouped into one large cluster with the *G. sulfurreducens* PpcA, while the remainder separated into 16 homolog clusters. Of the 58 cytochromes clustered by OMCL with *G. sulfurreducens* PpcA, 45 had also been designated as PpcA by bidirectional best-hit annotation. The remaining proteins annotated by best-hit as PpcA were scattered in other clusters by OCML, with no clear pattern for PpcB–E.

**Fig 3:**
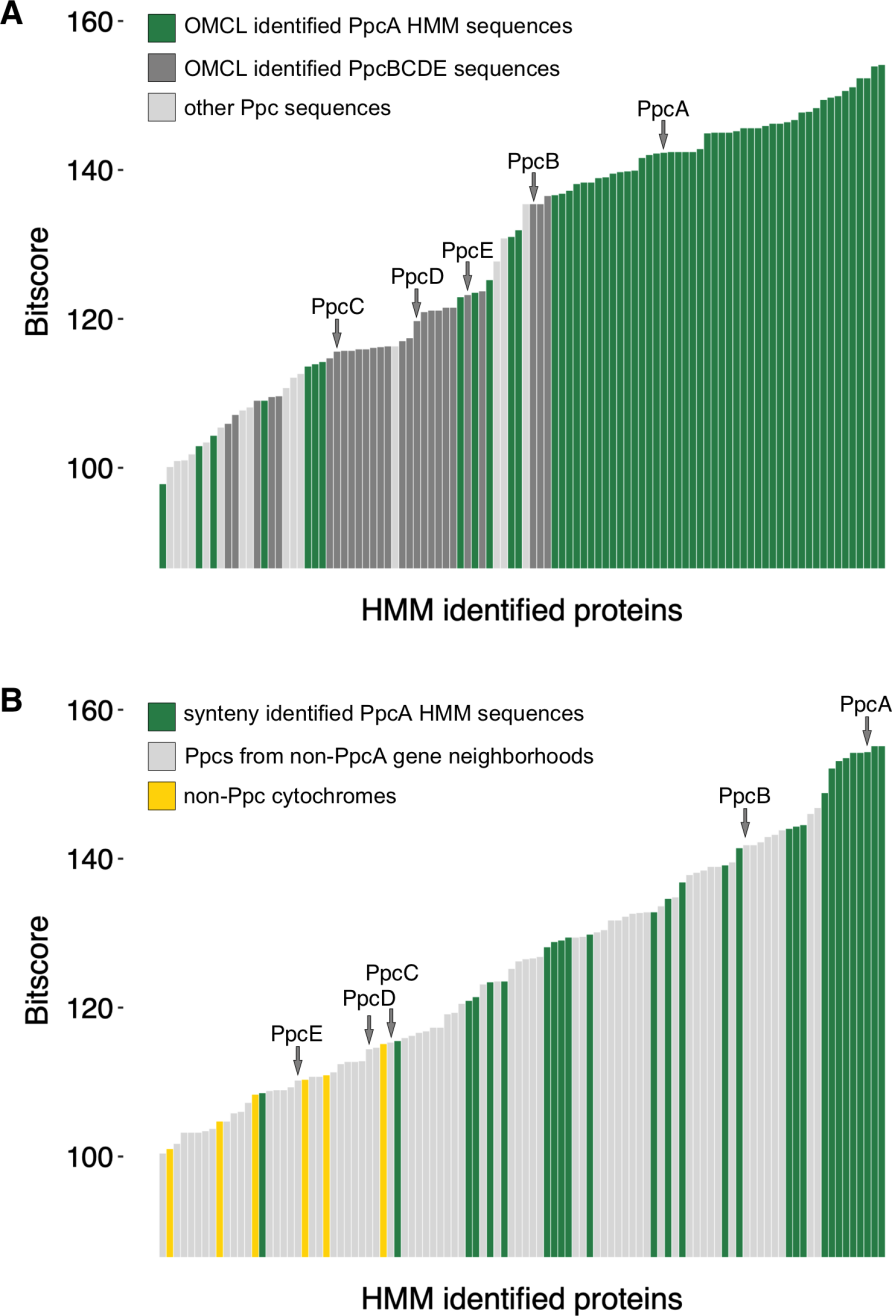
Neither homology nor synteny-based HMMs of PpcA can distinguish PpcA from other Ppc paralogs. (**A**) Results from the homology-based HMM, using PpcA sequences clustered by OCML with *G. sulfurreducens* PpcA. Sequences designated PpcBCDE were clustered by OMCL with *G. sulfurreducens* PpcB, C, D, or E. (**B**) Results from the synteny-based PpcA HMM, using manually curated PpcA sequences. Ppc representatives from *G. sulfurreducens* PCA are designated by arrows.

We then asked if signature residues or motifs could allow more sensitive comparisons. For example, sequences from the 58-member OMCL PpcA cluster were aligned and used to construct an HMM. Using the pipeline FeGenie, which utilizes user-supplied HMMs and bitscore thresholds, we queried the original set of 33 genomes, accepting any hit over an intentionally low bitscore of 100 (22). This “OMCL PpcA-cluster” HMM retrieved 75 sequences (Fig. 3A). While most sequences used to construct the HMM scored higher than other sequences, there was no obvious bitscore cutoff that could separate members of the original PpcA pool from non-members. In fact, sequences clustering with *G. sulfurreducens* PpcB or PpcE scored very well using the PpcA HMM in the FeGenie pipeline, as did members of variant triheme non-Ppc family proteins lacking any *G. sulfurreducens* homologs.

In other words, an HMM constructed out of an aligned cytochrome collection, which was supposed to only identify that subset, instead would have named all paralogs within the *G. sulfurreducens* genome PpcA. This did not appear to relate to the physiological function or evolutionary history of these proteins.

As we previously noted, there is conserved synteny around some *ppc* genes, especially in strains closely related to *G. sulfurreducens*. Based on this synteny, we tried a manual approach to creating an authentic PpcA collection that could train an HMM (Fig. 3B). We hypothesized that synteny-confirmed sequences would provide a set of authentic PpcA sequences with shared ancestry. Such a set should have the best chance of identifying signature residues. Twenty-eight sequences were identified for this synteny-verified PpcA HMM.

Using the FeGenie pipeline, the HMM built from the careful synteny-based collection retrieved an even wider, and even less accurate, collection of cytochromes. This approach, designed to only identify authentic *ppcA* genes, scored *G. sulfurreducens* PpcB higher than 16 of the verified by synteny PpcA sequences from other genomes. Non-*ppcA* homologs within the *G. sulfurreducens* genome (PpcB–E) also scored well (Fig. 3B). This HMM retrieved cytochromes not even related to the Ppc family. Once again, there was no obvious bitscore cutoff that could be programmed into FeGenie to allow discrimination of PpcA from homologs separated into distinct clusters by OMCL or which had been annotated as non-PpcA cytochromes during annotation with Prokka.

This evidence suggests triheme cytochrome sequences do not maintain enough global or local sequence conservation for standard tools to provide reliable annotation outside of belonging to a triheme family. Whether it was standard BLAST best-hit, a more robust ortholog cluster algorithm, or manually curated HMMs using authentic homologs identified by gene synteny, each approach produced different and ambiguous results. Any of these methods would be useful for identifying triheme cytochromes mostly belonging to the Ppc family. None should be used for assigning orthology or transferring a true name like PpcA or PpcB.

One explanation for the failure of sequence-based methods in this example is that the Ppc family of cytochromes are “peptide-minimized” (23). These ~9–10 kDa/3 heme proteins are near the lower limit of how much peptide is needed to fold around and coordinate three hemes. Most of the evolutionary pressure may be focused on maintaining the heme fold, with few residues involved in physiologically relevant surface interactions. This is not just a feature of small cytochromes, as many larger multiheme cytochromes have a similar peptide-minimized ratio, such as the ~70 kDa/26 heme GSU2495, 32 kDa/12 heme ExtA, and 25 kDa/8 heme OmaB. Based on this assumption, annotation of such multiheme cytochromes may not be possible with such techniques, while larger cytochromes with a higher amino acid:heme ratio are more likely to contain useful information.

### Can we identify OmcS nanowires?

The *Geobacter* OmcS cytochrome that polymerizes into an extracellular nanowire poses a duplication problem similar to the Ppc cytochromes. In *G. sulfurreducens,* there are three hexaheme cytochromes related to OmcS (GSU2504). Two of these, OmcT (GSU2503), and an OmcS-like unnamed cytochrome (GSU2501), are encoded within an operon with *omcS*, while *omcJ* (GSU0701) is elsewhere. To date, the functions of *G. sulfurreducens* OmcS homologs are unknown, and only OmcS is known to form a wire, making it important to differentiate OmcS from its relatives (16). To capture all possible homologs, we collected all hexa-heme protein sequences within 10% of the *G. sulfurreducens* OmcS length from our 33-genome set. Unlike PpcA, where every genome contained multiple homologs, only 12 genomes had any cytochromes related to OmcS. The presence or absence of an *omcS*-like gene in a genome did not correlate with species phylogeny, providing evidence that this group is subject to loss and gain.

**Fig 4:**
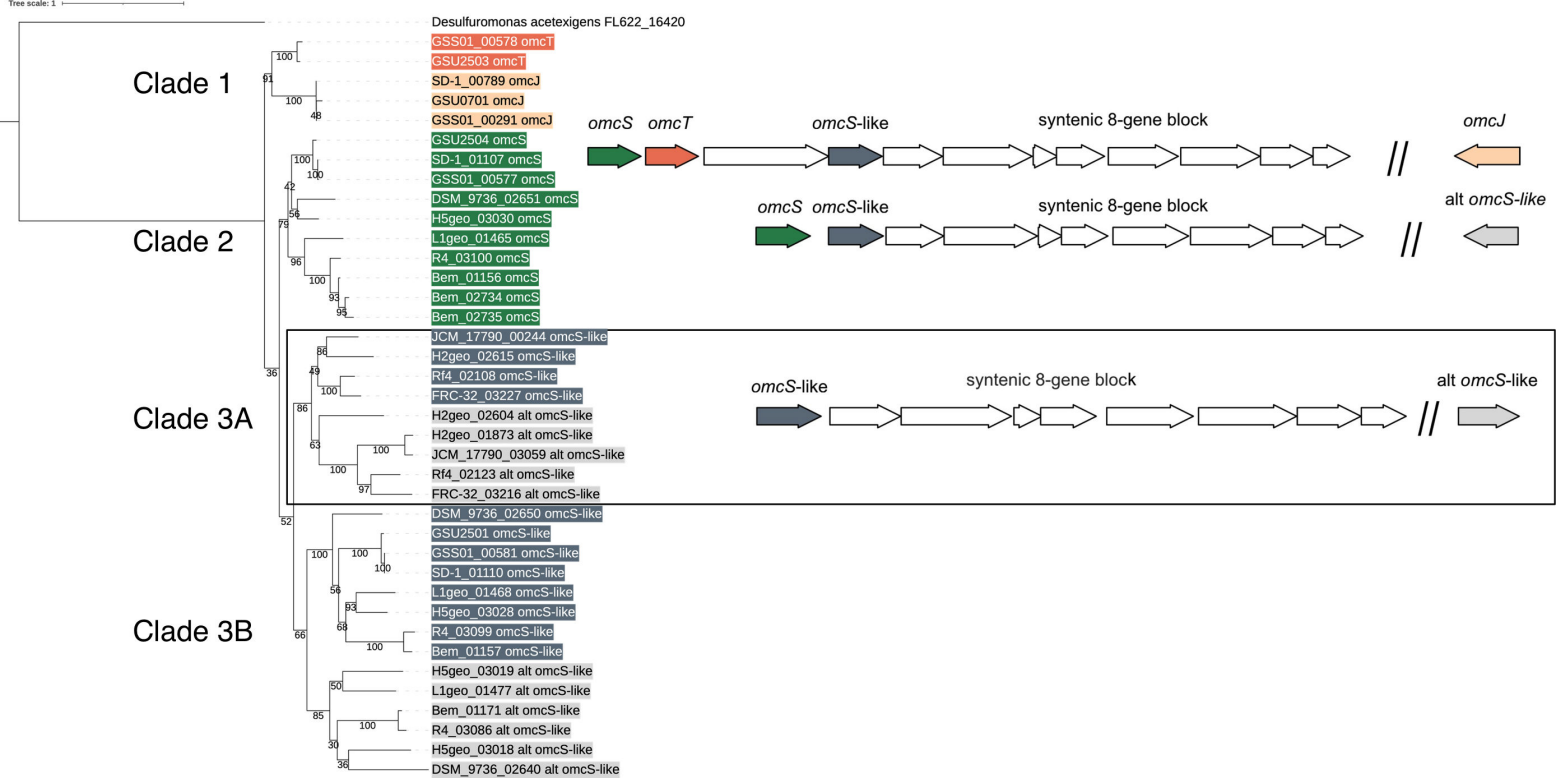
Phylogeny and gene synteny of *Geobacter* genes homologous to *G. sulfurreducens omcS*. Gene neighborhoods are color-coded to correspond to the gene phylogeny. The top gene neighborhood is found in *G. sulfurreducens*, *G. anodireducens*, and *G. soli*, with genes from Clades 1 and 2. *G. soli* and *G. anodireducens* have identical *omcT* sequences, so *G. anodireducens omcT* was removed from analysis. The middle gene neighborhood is found in *G. bemidjiensis*, *G. bremensis*, *G.* sp. DSM_9736, *G.* sp. H5geo, and *G.* sp. L1geo, with genes from Clades 2 and 3B. The boxed gene neighborhood is found in *G. daltonii*, *G. luticola*, *G.* sp. H2geo, and *G. uraniireducens*, with genes exclusively from Clade 3A.

A gene phylogeny of this *omcS* collection showed evidence of multiple independent duplication events (Fig. 4). For example, *G. sulfurreducens omcS*, *omcT,* and *omcJ* appear to have arisen from an ancestral hexaheme cytochrome, with *omcT* arising more recently. The phylogeny of these *omcS*, *omcT,* and *omcJ* copies is similar to that of a species tree of the strains containing these genes (Fig. 4, Clades 1 and 2). In *G. bemidjiensis*, instead of containing *omcT* and *omcJ*, there are instead three copies of *omcS* that are more closely related to each other than to any other homolog, suggesting another series of duplication and loss events. It is not clear whether each new *G. bemidjiensis omcS* still encodes a nanowire subunit (Fig. 4, Clade 2).

Cytochromes encoded by the *omcS*-like hexaheme GSU2501 that usually occurs near *omcS* do not cluster with *G. sulfurreducens omcS*, suggesting these diverged long ago. All genomes with this *omcS*-like GSU2501 homolog have an eight-gene block downstream (*G. sulfurreducens* GSU2500–GSU2493) recently linked to OmcS nanowire protein maturation and secretion (24). Not all 12 genomes containing the eight-gene “nanowire secretion” cluster encode a *G. sulfurreducens omcS*, but all encode the GSU2501 hexaheme cytochrome. This suggests GSU2501 could be ancestral to OmcS, and either forms an uncharacterized nanowire, or this cluster performed a secretion function that was later adopted for nanowire production when OmcS arose. Overall, hexaheme OmcS and its homologs are encoded in diverse patterns (Fig. 4): (i) an operon containing *omcST* and the GSU2501 *omcS*-like gene (sequences in Clades 1 and 2) *omcS* and a GSU2501 *omcS*-like gene (sequences in Clades 2 and 3B) or (ii) only a GSU2501 omcS-like gene upstream of the eight gene block (sequences in Clade 3A). Further, additional duplications remain unique to individual species, such as the hexaheme cytochromes elsewhere on the *G. bemidjiensis* genome.

**Fig 5:**
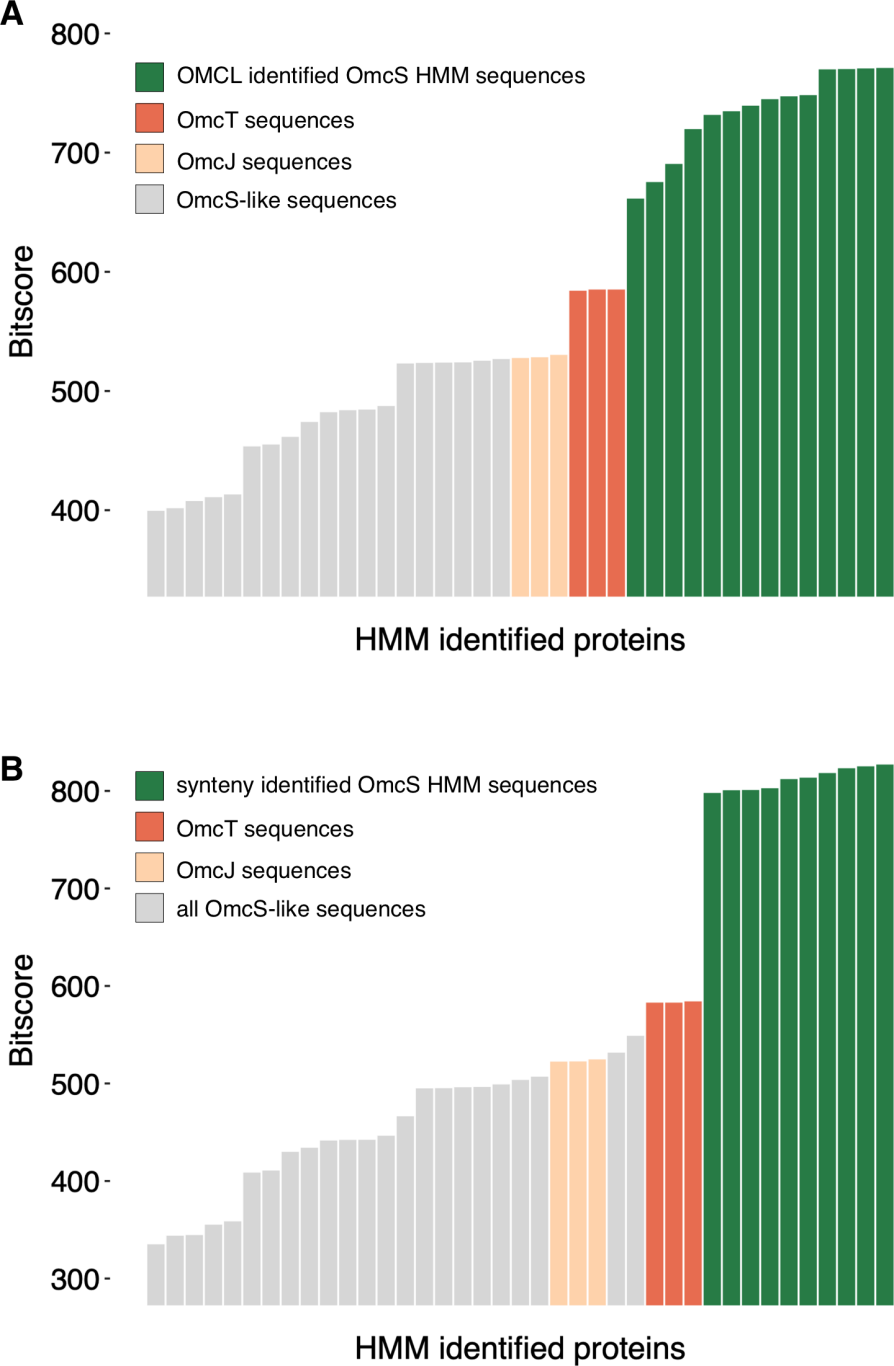
Homology and synteny-based OmcS HMMs are more successful at distinguishing OmcS from related 6-heme homologs. (**A**) Results from the homology-based OmcS HMM built from OMCL-clustered OmcS. (**B**) Results from the manually curated synteny-based OmcS HMM.

Like our efforts with PpcA, we tested whether homology-based or gene synteny-based alignments could identify “authentic” OmcS homologs or create HMMs capable of distinguishing these different hexaheme cytochrome families. For the homology-based identification, a small get_homologs cluster containing a subset most closely related to *G. sulfurreducens* OmcS was used to create an HMM. Unlike the results for the triheme cytochromes, the small number of input sequences used to create the HMM generated the highest scores and were more distinct from OmcT, OmcJ, and other OmcS-like sequences (Fig. 5A).

When OmcS sequences were more carefully selected using synteny, the resulting HMM-based identification was even more restrictive, clustering OmcT or OmcJ with the GSU2501 OmcS-like sequences (Fig. 5A). Together, these results suggest that, unlike with Ppcs, there may be signatures within very close relatives sufficient to distinguish OmcS from OmcT and OmcJ or other hexaheme cytochromes. We stress that such HMMs are likely only useful when searching within a genus and would lack the resolution needed to detect authentic OmcS nanowires in other genera.

OmcS and its homologs have higher kDa/heme ratios, which indicates there may be residues at active protein-protein interaction sites in addition to their heme-binding regions. Lacking clear information about which subunits form nanowires or catalyze other functions, sequence selection for HMM creation remains a highly manual process. In a case like *G. bemidjiensis*, recent duplications have created homologs that could not be distinguished by our methods without new functional information. The fact that only a few genomes contain OmcS, while others have multiple duplications, and that occasional genomes have hexaheme cytochromes of unknown function again highlights how these proteins appear to be rapidly changing.

### Is there evidence for greater mutation rates in *Geobacter* cytochromes?

The continuum of low bitscores from our alignments and HMMs suggested that the sequences of multiheme cytochromes changed faster than the average protein. To test this hypothesis, we performed mutation rate analysis on curated cytochrome homologs from our *Geobacter* genome set, as well as a set of *Shewanella* genomes.

In this analysis, the ratio of nonsynonymous nucleotide changes (dN) to synonymous nucleotide changes (dS) acts as a measure of the mutation rate over time. Since synonymous mutations are less affected by selective pressures than nonsynonymous mutations, they can act like a clock, normalizing comparisons between genes separated by different amounts of evolutionary time (25).

As both types of mutations should occur during DNA replication at the same rate, a dN/dS ratio of 1 would occur with no selection. If the protein sequence undergoes selection to retain the same residues, nonsynonymous mutations should not remain fixed in the population and would show a slower rate of amino acid changes relative to the baseline synonymous mutation rate. A selection to remain the same manifests as a low dN/dS ratio. Selection for the protein to adapt, or a lack of selection allowing drift, would cause the dN/dS to increase. This metric has the benefit of standardizing comparisons between closely related and distant species, assuming their baseline DNA mutation rate is the same.

In these analyses, dN and dS values were obtained by comparison of a reference gene in *G. sulfurreducens* PCA to its verified homolog in another *Geobacter* genome. *Shewanella oneidensis* MR-1 was used as the reference for *Shewanella*. Briefly, 33 *Geobacter* species and 39 *Shewanella* species were analyzed to obtain homolog clusters (Table S1). Because of problems described earlier with highly duplicated cytochromes such as Ppc or OmcS cytochromes, we eliminated genes that contained unresolvable paralogs in each genome. Clusters containing a reference PCA or MR-1 sequence were manually curated as follows: (i) the homolog sequence length had to be within 20% of the reference protein; (ii) the number of heme-binding motifs had to be ±2 of the reference; (iii) if a cluster contained two or more homologs from the same genome, the correct cytochrome homolog had to be manually verified, or else the sequences were removed from further analysis.

Pangenome matrices derived from all *Geobacter* or *Shewanella* genomes were also used to identify conserved genes present in all genomes, designated as core genes. Protein sequences aligned with Clustal Omega were then used to codon-format corresponding nucleotide sequences so that mutation rates for each homolog could be obtained by pairwise comparisons of each sequence using the KaKs Calculator (26, 27).

**Fig 6:**
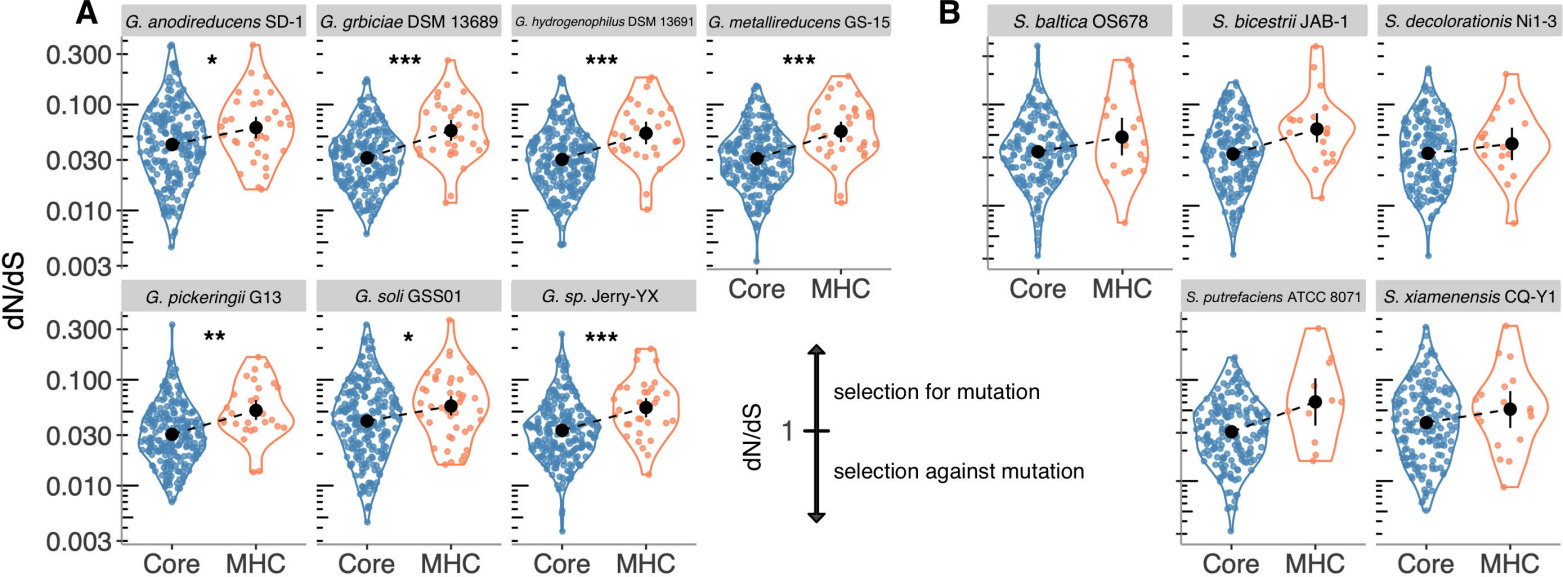
Comparison of core vs multiheme cytochrome mutation rates in *Geobacter* and *Shewanella*. Higher values represent faster rates of nonsynonymous amino acid replacements. (**A**) Core vs multiheme cytochrome mutation rates from genomes in the *Geobacter sulfurreducens* clade. (**B**) Core vs multiheme cytochrome mutation rates from genomes in the *Shewanella oneidensis* clade. Significant differences are designated by asterisks: *P < 0.05, **P < 0.001, and ***P < 0.0001; ns, not significant.

The results of these mutation rate analyses show that, for the majority of analyzed *Geobacter* genomes (25/32), mutation rates are significantly higher for cytochrome genes than core protein-encoding genes (Fig. 6A; Fig. S1). As many cytochromes with high mutation rates (high dN/dS ratios) had to be removed from our final analysis due to the potential for erroneously comparing paralogous sequences, the magnitude of this mutation rate is likely underestimated.

The results for *Shewanella* also showed 16/38 genomes had statistically significant differences (Fig. 6B; Fig. S2). As *Shewanella* do not have as many cytochromes as *Geobacter,* insufficient data may contribute to the lack of statistically significant differences between core and cytochrome genes in *Shewanella*. Notably, five *Geobacter* genomes that did not have statistically significant differences between core and multiheme genes also were strains with the fewest multiheme cytochromes (<22; Fig. S1).

While direct comparison of *Geobacter* dN/dS rates to *Shewanella* dN/dS rates is not possible as they do not have any cytochromes in common, mutation rates were highest in *Geobacter* cytochromes. Although many *Geobacter* genomes have since been assigned to other Geobacteraceae genera, all genes in this analysis were standardized to the synonymous mutation rate. This means the enhanced mutation rates in our data set are not due to larger phylogenetic distances between *G. sulfurreducens* and genomes reassigned to new genera but are inherent characteristics of these proteins.

**Fig 7:**
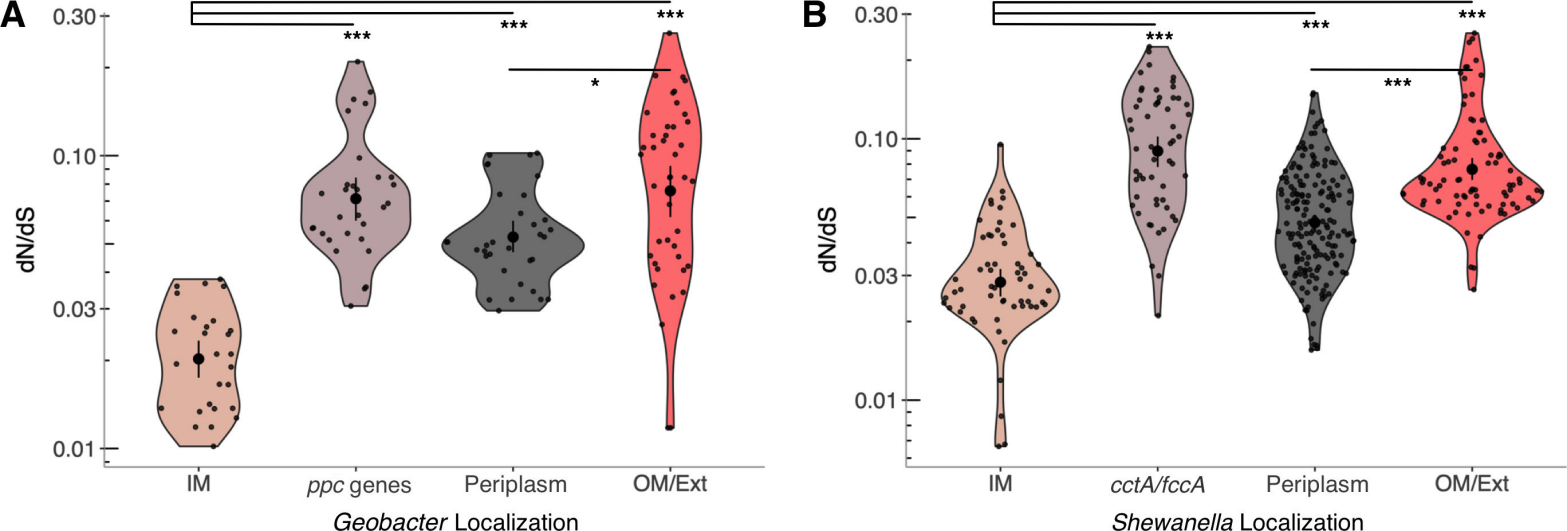
Comparison of multiheme cytochrome mutation rates in different cell locations in *Geobacter* and *Shewanella*. (**A**) Mutation rates for orthologs in genomes within the *Geobacter sulfurreducens* clade. IM: *imcH, cbcL*, and *cbcBA*; *ppc: ppcABCDE*; periplasm: *nrfA*, *nrfH*, *extA*, *omaC*, and *extK*; OM/ext: *omcE*, *omcS*, *omcZ*, *pgcA*, *extD*, *extC*, and *omcC*. (**B**) Mutation rates for *Shewanella* genomes. IM: *cymA* and *torC*; periplasm: *nrfA*, *otr*, *sirA*, *mtrA*, *mtrD*, and *dmsE*; OM: *mtrC*, *mtrF*, and *omcA*.

While *Geobacter* cytochromes show greater mutation rates compared to other conserved proteins, we observed that mutation rates for cytochromes were not uniformly high. We hypothesized that the localization or function of cytochromes within the electron transport chain could be correlated with the mutation rate and reanalyzed dN/dS data to test this hypothesis. For *Geobacter*, in order to include carefully annotated periplasmic *ppc* genes in this analysis, we used data only from the *G. sulfurreducens* clade or *Geobacter* genus to use only closely related strains, where orthologs could be verified (Fig. 1). Analysis of dN/dS ratios showed three distinct groups in both the *G. sulfurreducens* and *Shewanella* data: (i) “low” mutation rates, typically exhibited by inner membrane cytochrome genes; (ii) “medium” mutation rates, exhibited by non-*ppc*/*fccA*/*cctA* periplasmic cytochrome genes; (iii) “high” mutation rates, exhibited by *ppc*/*fccA*/*cctA*, outer membrane, and extracellular cytochrome genes (Fig. 7).

Of interest is the fact that genes encoding periplasmic cytochromes that are part of larger porin-cytochrome complexes, as well as periplasmic cytochromes with strictly enzymatic functions, had lower dN/dS ratios compared to *ppc*, *cctA*, and *fccA* genes. PpcA, CctA, and FccA are presumed to be free-floating in the periplasm, and, with the exception of FccA, which is also a fumarate reductase, their functions are limited to electron transfer (28). Overall, our results indicate that the location or function of cytochromes can influence their evolutionary rate, making identification of some more reliable than others.

### The expanding cytochrome pangenome

During our analyses, we noted that *Geobacter* species have large cytochrome repertoires that undergo gain, loss, duplication, and greater mutation rates, so we asked whether a consensus repertoire can even be defined. For the 33 *Geobacter* genomes analyzed (Table S1), there was only one cytochrome with sequences in all genomes, the PpcA sequence, with the caveat that absolute identification of PpcA homologs remains questionable. Many of the analyzed genomes have been reassigned into new genera, meaning there are no cytochromes conserved across the roughly three genera analyzed in this work.

**Fig 8:**
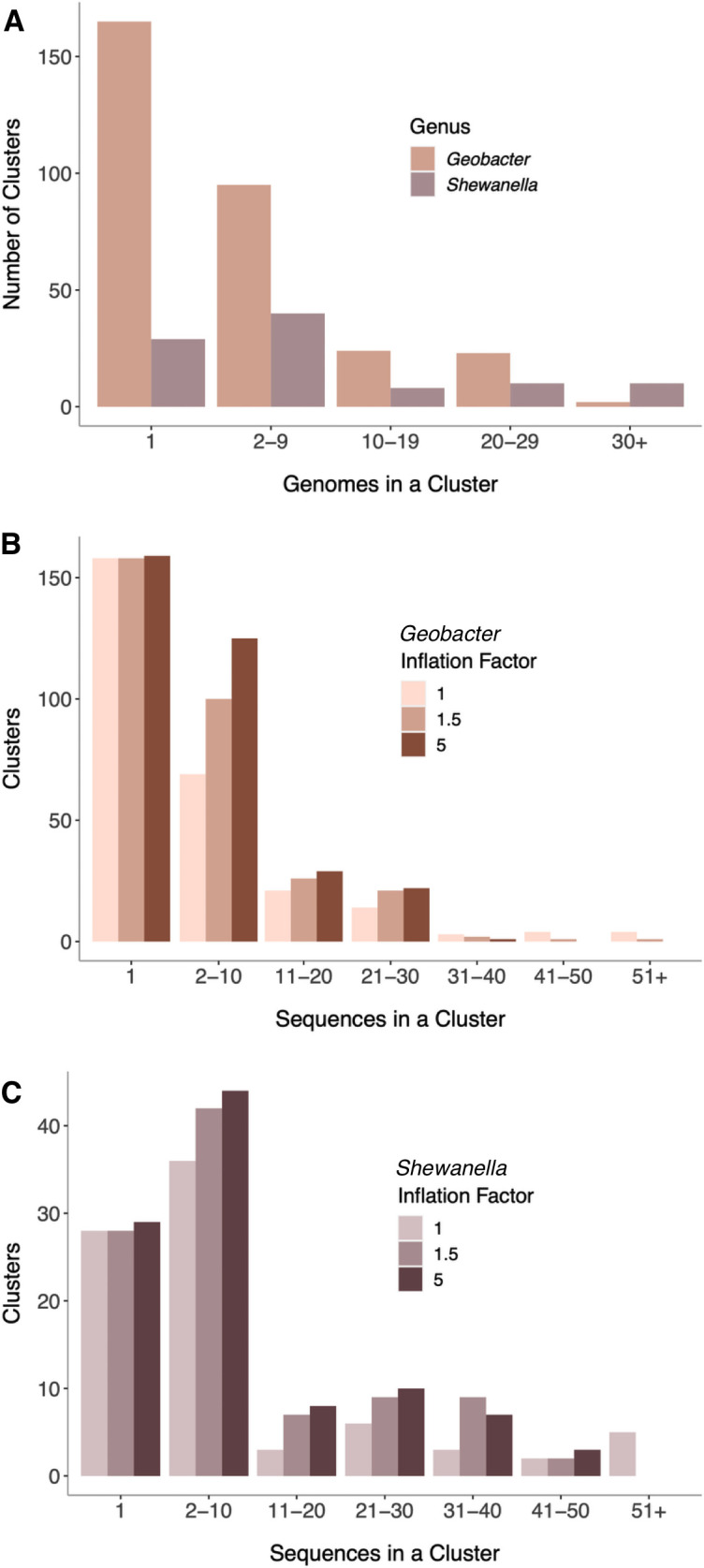
OMCL-based clusters of MHC sequences are dominated by clusters with very few members. (**A**) *Geobacter* cytochrome homolog clusters binned by the number of genomes containing a representative from a cluster. Over 150 cytochromes were found that lacked a homolog in any other *Geobacter* genome. (**B**) Reducing homolog-calling stringency does not affect the number of *Geobacter* homolog clusters containing only one member. (**C**) Reducing homolog-calling stringency does not affect the number of *Shewanella* homolog clusters containing only one member. Legend: F1, lowest stringency; F1.5, default stringency; F5, highest stringency.

Across the *Geobacter* genomes, a large number of multiheme cytochromes were found only one time. In other words, 53% of the cytochrome clusters were represented by only a single sequence, which occurred in only one genome. Cytochromes found in only 2–9 genomes represented another large percentage (31%). Only 16% of cytochromes contained homologs in more than 10 genomes (Fig. 8A). This suggested that not only are cytochromes evolving at rapid rates but also that each *Geobacter* genome contains a significant number of novel cytochrome sequences.

As this result could be an artifact of the stringency of our homolog calling, we repeated the analyses using the highest and lowest stringency settings. Changing stringency had a negligible effect on the number of unique multiheme cytochromes identified. As expected, at lower stringency, more paralogs collapsed together into larger clusters, grouping most PpcA-like or OmcS-like sequences into a single group, but the number of single-cytochrome clusters remained unchanged (Fig. 8B). This supported the conclusion that while genomes have many cytochromes due to duplication, the high number of novel multiheme cytochromes, in particular the number of unique singletons found in each genome, is not an artifact of the clustering algorithm.

The same analyses in *Shewanella* found that the proportion of cytochromes shared across the genus was greater than in *Geobacter* (Fig. 8A and C). Twenty percent of homolog clusters were found in more than half of the genomes analyzed (Fig. 8A). This could partially be due to the analyzed *Shewanella* genomes being more closely related. However, we found that clusters containing only one sequence constituted 30% of the total pool, as compared to 53% for *Geobacter* (Fig. 8C). The lower proportion of singleton clusters, in combination with the lower absolute numbers of cytochromes, shows that the *Shewanella* multiheme cytochrome pool is less diverse than in *Geobacter* and related genera.

This raised the question of what level of sampling of *Geobacter* and *Shewanella* genomes would saturate the pool of cytochromes in their respective groups. For this analysis, we calculated the phylogenetic distance of each genome from the reference genome (*G. sulfurreducens* PCA or *S. oneidensis* MR-1) using a whole-genome maximum likelihood approach (29). We then conducted clustering analyses by adding genomes one by one, in order of the least to greatest phylogenetic distance from the reference. If sampling is approaching saturation, adding a new genome should not add any unique cytochromes. The number of clusters discovered as each new genome is added should plateau.

**Fig 9:**
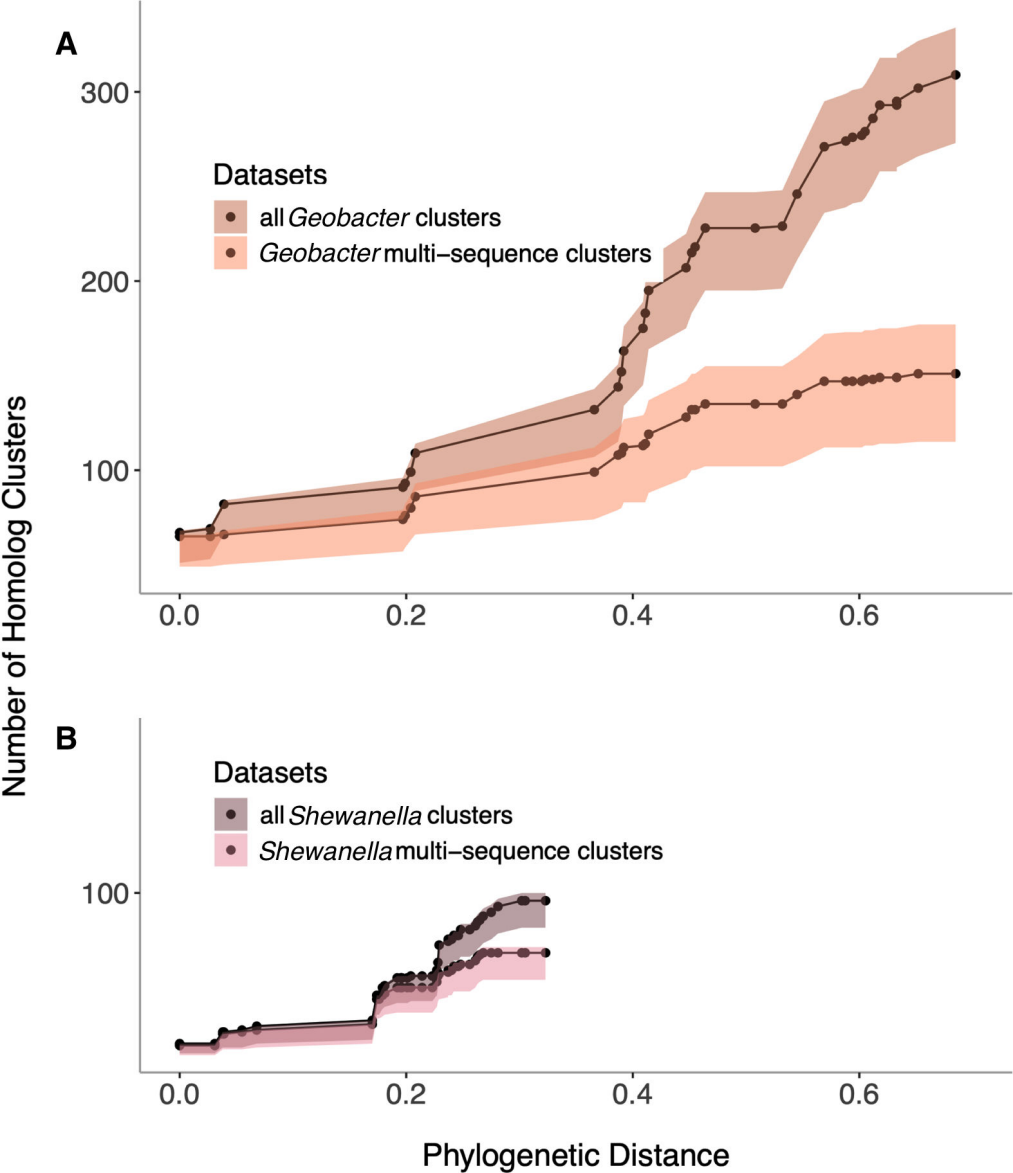
The number of unique multiheme homolog clusters continues to increase with the addition of new genomes. Analysis showing the number of total cytochrome clusters as new *Geobacter* (**A**) and *Shewanella* (**B**) genomes are added. Counting only clusters with sequences found in two or more genomes shows how the increase in new families is driven by the presence of novel multiheme cytochromes lacking any homolog in any genome.

For *Geobacter*, the number of novel clusters continued to increase with the addition of each new genome, while clusters in *Shewanella* were beginning to taper (Fig. 9A). Given that half of the cytochromes in our *Geobacter* analysis were unique, lacking any homologs in other genomes, it is not surprising that we did not see saturation. When we removed these unique “singleton” sequences, the number of cytochromes shared by two or more genomes did begin to reach saturation (Fig. 9B). These results suggest that there are multiple pools of cytochromes with different evolutionary trajectories. Some, such as the inner membrane cytochromes, are mostly vertically inherited and have slower rates of evolutionary drift. Another pool, like nanowire or porin-cytochrome cytochromes, is less well conserved, with any representative only being found in 30%–50% of genomes. These are constantly being lost, gained, duplicated, and exchanged between organisms. The most enrigmatic population seems to be frequently horizontally acquired and lost, forming a large reservoir of cytochromes unqiue to each genome.

## DISCUSSION

The reductases of most anaerobic energy generation strategies are well conserved and easy to identify. Metagenomes can quickly be annotated to predict if organisms participate in cycling of acceptors such as nitrate, nitrite, sulfate, polysulfide, arsenate, selenate, chlorinated compounds, DMSO, TMAO, and carbon dioxide. Since the first genomes of dissimilatory metal-reducing bacteria were sequenced, it has been clear that something about the proteins central to their metabolism was different.

Many key cytochromes essential to extracellular electron transfer are now known in model strains. When annotating future genomes and transferring this growing body of functional information, the assumption is made that, like other enzymes, these sequences and their functions are conserved. However, the presence of abundant paralogs, lack of cytochromes conserved between species, and evidence for increased mutation rates raises questions about whether information from one strain should be mapped in automated bioinformatic pipelines (1, 10). For example, this study found that in families like PpcABCDE and OmcSTJ, the identification or assignment of function across broader *Geobacter* genera faces significant hurdles.

While duplications and paralogs are known issues in metal-reducing bacteria, the mutation rates of these proteins remained largely unexplored. A high mutation rate was reported within multiheme cytochrome sequences when *G. sulfurreducens* PCA was compared to the *G. sulfurreducens* variant KN400. While these strains had 96% of coding sequences in common and 72% of single-nucleotide differences were synonymous mutations, 25 orthologs had notably high rates of nonsynonymous changes. This pool was significantly enriched in *c*-type cytochromes (30). In our extended analysis*,* this increased cytochrome mutation rate was a consistent trait across *Geobacter*.

While multiheme cytochromes had higher mutation rates in both *Geobacter* and *Shewanella* genomes, we found this mutation rate to be further correlated with cytochrome location. Inner membrane protein-encoding genes have the lowest mutation rates, especially in their membrane domains, which may be due to additional sequence constraints imposed by bioenergetic coupling, reacting with the quinone pool, and interacting with periplasmic partners. These quinone oxidoreductases, such as ImcH, CbcL, CbcBA, and CymA, remain among the best markers for identifying putative extracellular electron transfer in metagenomic studies due to their slower mutation rates and lower frequency of loss from genomes.

In contrast, genes from outer surface cytochromes demonstrate the highest mutation rates. A basis for these higher rates was suggested by the structures of cytochrome nanowires, where there is a high degree of structural alignment between hemes. For example, the four hemes of OmcE and four hemes in OmcS are superimposable, even though these nanowires lack any sequence similarity (15, 31). Both proteins lack substantial amounts of secondary structure, allowing mutations, insertions, and deletions to easily occur in loops and coils, so long as they do not perturb the alignment and coordination of hemes (15, 31). If the primary selective pressure on a cytochrome is to maintain the same heme coordination, much more variation is possible. In the case of surface-exposed cytochromes, recent data showing that the *Shewanella* OmcA cytochrome is a phage target indicates that pressure for phage resistance may create additional selection for amino acid changes in extracellular cytochromes (32).

Unlike enzymes with highly evolved active sites, where specific residues are essential, multiheme cytochromes may be able to perform the same job with vastly different amino acids, providing they (i) have the correct heme coordination; (ii) have the correct redox potential; (iii) have a surface charge that lets them get close enough to their partner for electron transfer to occur. In the case of *G. sulfurreducens*, successfully transferring electrons across the periplasm only requires sufficient expression of any one of the five Ppc homologs, and this function can even be performed by a cytochrome typically secreted to the outer surface (10). The fact that increased cytochrome mutation rates are also seen in *Shewanella* suggests that such selective pressure and promiscuous behavior are not unique to *Geobacter*. In this case, periplasmic cytochromes will provide unreliable markers for extracellular electron transfer and be consistently misannotated.

Unlike most reductases that form the basis for most anaerobic respirations, whose presence or absence can signal an ability, the cytochromes of *Geobacter* represent an opposite situation. The number of cytochromes per genome is high, but a low percentage of cytochromes shared between any two strains means there are few to use as markers. Every sequenced genome reveals new cytochromes not present in any other strain, raising the question of where these unique proteins are coming from. Are these unique cytochromes the result of such rapid evolution that we can no longer recognize similarity with their ancestors or is there an abundance of unique proteins being exchanged via horizontal gene transfer from species yet to be isolated?

A similar problem of abundant unclassifiable cytochromes was raised in a recent survey of a much larger collection of *Geobacter-*related genomes (33). In this survey of all available *Desulfuromonadia*, the inability to discern paralogs required treating all hexaheme OmcS, OmcT, and OmcJ-like cytochromes as similar. This allowed finding whether a genome did or did not have between 0 and 5 *omcS*-like genes, but prevented deduction of whether nanowire-like functions were present. In addition, even when using a MMSeqs-based approach less stringent than what was applied in our work, nearly half of the *Desulfuromonadia* 4,716 cytochromes analyzed had no known homologs in other databases.

While clustering algorithms parse a large percentage of cytochromes as unique in sequence, they may not functionally be so. As shown in cytochrome nanowires, we may find that strict adherence to the primary sequence is not as important for function as it is for other types of proteins. Based on this evidence, cytochrome classification may need to be guided by a combination of local gene synteny and structural comparison being made possible by the emergence of Cryo-EM and AlphaFold-like tools. We may be able to shrink this growing universe of sequence variation by clustering according to common shapes, heme-packing motifs, and local organization patterns (34).

In conclusion, this study suggests caution when using annotation tools and raises new questions about the perplexing redundancy of multiheme cytochromes in *Shewanella* and *Geobacter*. Are increased mutation rates a result of a constant searching for more fit cytochromes, or are protein sequences simply drifting as they are not penalized as strongly as they are in enzymes? How much mutation can a multiheme cytochrome tolerate before its function changes? Are enhanced mutation rates unique to specific extracellular electron transfer-performing organisms, or are they a broader evolutionary trend in multiheme cytochromes as a class? What is the size and mechanism supporting this vast horizontal gene transfer pool? Answers to these questions will help address the severe limitations currently facing annotation of cytochrome-rich genomes and help in identification of new organisms capable of extracellular electron transfer.

## MATERIALS AND METHODS

### Selection and preparation of genomes for analysis

Genomes were obtained from NCBI in October 2021. We selected assemblies identified as “*Geobacter”* having at least 50× sequencing coverage and, if the genome was a metagenomic MAG, ≥3 MB to filter out low-quality or incomplete assemblies. For each *Shewanella* species, a strain with a complete genome was selected. *Geobacter* and *Shewanella* genomes having no MHCs were removed from further analysis. The *Geobacter sulfurreducens* PCA genome was manually annotated to reflect the current literature on cytochrome and cytochrome-related genes and then used as a reference to annotate our selected *Geobacter* genomes using Prokka (19). For *Shewanella* species, *Shewanella oneidensis* MR-1 was used as the Prokka reference. In addition, all genes encoding MHCs were annotated with the number of hemes in case any lacked homologs in the reference strain and to create the full set for pangenome analysis.

### Phylogenetic trees

Maximum-likelihood trees for genomes were constructed with PhyloPhlAn using the amino acid supermatrix configuration file, which uses the PROTGAMMA-LG model of amino acid substitution, and the PhyloPhlAn database of 400 universal marker genes (29). For the *Geobacter* tree, the --medium diversity setting was used as the selected genomes are from several genera in the family Geobacteraceae. *Desulfuromonas acetoxidans* DSM 684 and *Pelobacter carbinolicus* DSM 2380, from the Desulfuromonadaceae family, were used as the outgroup. High diversity was used for the combined *Geobacter* (Desulfobacterota) and *Shewanella* (Pseudomonadota) tree. *Geothrix fermentans* DSM 14018 and *Acidobacterium capsulatum* ATCC 51196 from Phylum Acidobacteriota were used as the outgroup.

For alignment of gene sequences, the online MAFFT tool from EMBL-EBI was used with default parameters. Maximum-likelihood gene trees were constructed using RAxML 8.2.11 (pthread), using the GTRGAMMA or PROTGAMMA models, depending on whether DNA or protein was being analyzed (35, 36). RAxML was also used for bootstrapping, using rapid bootstrap analysis for protein-based genome trees and the PROTGAMMA-LG model. Both the *Geobacter* tree (Fig. 1) and the combined *Geobacter* and *Shewanella* tree (Fig. S3 and S4) converged after 50 replicates. Rapid bootstrap analysis for the *omcSTJ* tree was performed with convergence after 600 replicates.

### Homolog clustering

Homolog clustering was performed with the bioinformatic tool get_homologs (20, 21, 37–44). Unless otherwise specified, genomes were analyzed using a reference genome and the OMCL algorithm, with all other settings as default. Pangenome matrices were obtained using the OMCL algorithm.

### Cytochrome diversity by phylogenetic distance

To calculate the number of new cytochromes identified with increasing phylogenetic distance from a reference genome, data from both get_homologs and a maximum-likelihood *Geobacter* and *Shewanella* tree were used. Distances between each genome and their respective reference were calculated using the *Geobacter* and *Shewanella* maximum-likelihood tree. Then, get_homologs was used to cluster proteins from genomes, adding genomes one at a time in order of distance. This was performed using inflation values (clustering stringency) of 1 (minimum stringency), 1.5 (default), and 5 (maximum stringency). The number of new cytochrome clusters per genome added was then calculated and plotted against phylogenetic distance.

### HMM construction and FeGenie

Input sequences for HMMs were chosen by get_homologs clustering, Prokka-based annotation, or manually curated using synteny where indicated. Amino acid sequences were aligned using MUSCLE (45). HMMs were constructed using hmmbuild in the bioinformatic package HMMER (43). Alignments and HMMs were manually added to FeGenie, along with our manually selected bitscore cutoff, to identify sequence hits (22).

### Mutation rate analysis

Input sequences were obtained from get_homolog cytochrome and “core” gene and protein homolog clusters. To ensure that comparisons were between homologs, cytochrome clusters were curated as follows: (i) the number of heme-binding motifs must equal the reference ± 2; (ii) the protein sequence length must be within 20% of the reference; (iii) multiple sequences from the same genome within a cluster must be verified to be a duplication or the correct homolog identified, or else the sequences will be removed from further analysis. Protein sequences from homolog clusters were aligned using Clustal Omega (27). Sequences in gene homolog clusters were then codon-formatted with their corresponding amino acid alignment files. Files with codon-formatted sequences were then converted to axt format using a custom script. Mutation rates for each homolog cluster were obtained by pairwise comparison of each sequence with the cluster’s PCA or MR-1 sequence using the modified Yang-Nielsen method with the KaKs Calculator bioinformatic tool (26, 46).

### Statistical analyses

All statistical analyses were performed in R (34). For all groups of dN/dS data, normality was tested using the Shapiro-Wilks test. Variance for normal data was calculated with the F-test. Cytochrome dN/dS values often had a non-normal distribution, with core dN/dS values being normal. In the former case, these data were log-transformed before performing two-sample *t*-tests, or the Welch’s *t*-test, in the case of unequal variance. Where both sets of dN/dS values had non-normal distributions, the Wilcoxon rank-sum test was used. For comparison of cytochrome dN/dS values by localization, the Kruskal-Wallis test was used with Dunn’s *post hoc* test.

## SUPPLEMENTAL MATERIAL

Supplemental Material mbio.03394-25-s0001.pdf Table S1; Figures S1-S4.

ASM does not own the copyrights to Supplemental Material that may be linked to, or accessed through, an article. The authors have granted ASM a non-exclusive, world-wide license to publish the Supplemental Material files. Please contact the corresponding author directly for reuse.
