# Supplementary material for: Increased mutation rates and diversity are dominant features of Geobacter multiheme cytochromes
Source: mBio. 2026 Feb 17;17(3):e03394-25. doi: 10.1128/mbio.03394-25 (PMC12977616; doi:10.1128/mbio.03394-25)
Supplement: Supplemental Material — Table S1; Figures S1-S4. [file mbio.03394-25-s0001.pdf]

Supplemental Table & Figures

Genomes	Genbank Accession Num	Isolation source
Geobacter anodireducens SD-1	GCA_001628815.1	microbial fuel cell biofilm
Geobacter argillaceus ATCC_BAA_11	GCA_007830735.1	kaolin clays, USA
Geobacter bemidjiensis Bem	GCA_000020725.1	freshwater sediment, USA
Geobacter bremensis R4	GCA_014218275.1	unknown
Geobacter chapellei DSM_13688	GCA_018531195.1	freshwater sediment, USA
Geobacter daltonii FRC-32	GCA_000022265.1	hydrocarbon contaminated sediment, USA
Geobacter grbiciae DSM_13689	GCA_018531165.1	freshwater sediment, USA
Geobacter hydrogenophilus DSM_136	GCA_018501225.1	hydrocarbon contaminated aquifer, USA
Geobacter lovleyi SZ	GCA_000020385.1	freshwater sediment, South Korea
Geobacter luticola JCM_17780	GCA_018476905.1	lotus field sediment, Japan
Geobacter metallireducens GS-15	GCA_000012925.1	freshwater sediment, USA
Geobacter pelophilus Drf2	GCA_002117975.1	freshwater sediment, Germany
Geobacter pickeringii G13	GCA_000817955.1	kaolin clays, USA
Geobacter soli GSS01	GCA_000816575.1	humic layer soil, China
Geobacter sp. Bin_37_3	GCA_009885845.1	freshwater sediment, Canada
Geobacter sp. DSM_2909	QAXR01000001.1	starch wastewater digester, France
Geobacter sp. DSM_9736	GCA_900187405.1	paddy soil, Italy
Geobacter sp. FeAM09	GCA_008330225.1	forest soil, USA
Geobacter sp. GAC1	GCA_003314535.1	digester fed methanogenic reactor, USA
Geobacter sp. H2geo	GCA_003574885.1	anode biofilm, USA
Geobacter sp. H5geo	GCA_009684525.1	anode biofilm, USA
Geobacter sp. H7geo	GCA_009684515.1	anode biofilm, USA
Geobacter sp. Jerry-YX	GCA_017338855.1	petroleum soil, China
Geobacter sp. L1geo	GCA_003574895.1	anode biofilm, USA
Geobacter sp. SVR	GCA_016865365.1	mine soil, Japan
Geobacter sp. UBA698	GCA_002298925.1	anode biofilm
Geobacter sp. UBA2189	GCA_002328035.1	tailing pond, Canada
Geobacter sp. UBA6151	GCA_002423105.1	wastewater, Canada
Geobacter sp. UBA9964	GCA_003490025.1	groundwater
Geobacter sp. UBA12467	GCA_003488785.1	groundwater
Geobacter sulfurreducens PCA	GCA_000007985.2	hydrocarbon contaminated sediment, USA
Geobacter thiogenes ATCC_BAA-34	GCA_900167465.1	contaminated subsoil, USA
Geobacter uraniireducens Rf4	GCA_000016745.1	contaminated subsoil, USA
Shewanella aestuarii PN3F2	GCF_011765625	*Perinereis linea* intestine
Shewanella algae RQs-106	GCF_009730655	activated sludge
Shewanella amazonensis SB2B	GCF_000015245	marine sediment, Amazon River delta
Shewanella avicenniae FJAT-51800	GCF_017354945	estuary sediment, China
Shewanella baltica OS678	GCF_000178875	free-living marine, Baltic Sea
Shewanella benthica DB21MT-2	GCA_900476435	marine sediment, Mariana Trench
Shewanella bicestrii JAB-1	GCF_002216875	bile, *Homo sapiens*
Shewanella carassii TUM17387	GCF_019670705	*Homo sapiens*
Shewanella chilikensis DC57	GCA_011106835	corroded pipe, Australia
Shewanella cyperi FJAT-53726	GCF_017354925	*Cyperus malaccensis* rhizosphere*, China*
Shewanella decolorationis Ni1-3	GCA_007923045	electroplating wastewater sludge
Shewanella denitrificans OS217	GCF_000013765	Baltic sea
Shewanella dokdonensis DSM_23626	GCF_018394335	seawater, South Korea
Shewanella donghaensis LT17	GCF_007567505	deep sea sediment, Sea of Japan
Shewanella eurypsychrophilus YLB-0	GCF_007004545	deep sea sediment, Indian Ocean
Shewanella frigidimarina NCIMB_400	GCF_000014705	seawater, North Sea
Shewanella glacialimarina TZS-4	GCF_020511155	sea ice, Baltic Sea
Shewanella halifaxensis HAW-EB4	GCF_000019185	marine sediment, Atlantic Ocean
Shewanella inventionis D1489	GCF_019931735	marine sediment, Pacific Ocean
Shewanella japonica KCTC_22435	GCF_002075795	seawater, Troitza Bay
Shewanella khirikhana TH2012	GCF_003957745	*Penaeus vannamei* hepatopancreas
Shewanella litorisediminis SMK1-12	GCF_016834455	tidal flat sediment, South Korea
Shewanella livingstonensis LMG_198	GCF_003855395	Antarctic water
Shewanella loihica PV-4	GCF_000016065	hydrothermal vent, Pacific Ocean
Shewanella marisflavi EP1	GCF_002215585	marine sediment, China
Shewanella maritima D4-2	GCF_004295345	seawater, South Korea
Shewanella oneidensis MR-1	GCA_000146165	freshwater sediment, Lake Oneida
Shewanella pealeana ATCC_700345	GCF_000018285	*Loligo pealei* nidamental gland
Shewanella piezotolerans WP3	GCF_000014885	deep sea sediment, Pacific Ocean
Shewanella polaris SM1901	GCF_006385555	brown alga, Arctic Ocean
Shewanella psychrophila WP2	GCF_002005305	deep sea sediment, Pacific Ocean
Shewanella psychropiezotolerans YLB	GCF_007197555	deep sea sediment, Indian Ocean
Shewanella putrefaciens ATCC_8071	GCF_016406325	butter
Shewanella sedimentimangrovi FJAT-	GCF_017354965	estuary sediment, China
Shewanella sediminis HAW-EB3	GCF_000018025	marine sediment, Atlantic Ocean
Shewanella vesiculosa M7	GCA_021560015	marine sediment, Antarctic coast
Shewanella violacea DSS12	GCF_000091325	deep sea sediment, Ryukyu Trench
Shewanella woodyi ATCC_51908	GCF_000019525	detritus, Alboran Sea
Shewanella xiamenensis CQ-Y1	GCA_019973655	oilfield wastewater, China

Table S1. Genomes used in bioinformatic analyses. All genomes and genome data were obtained from NCBI.

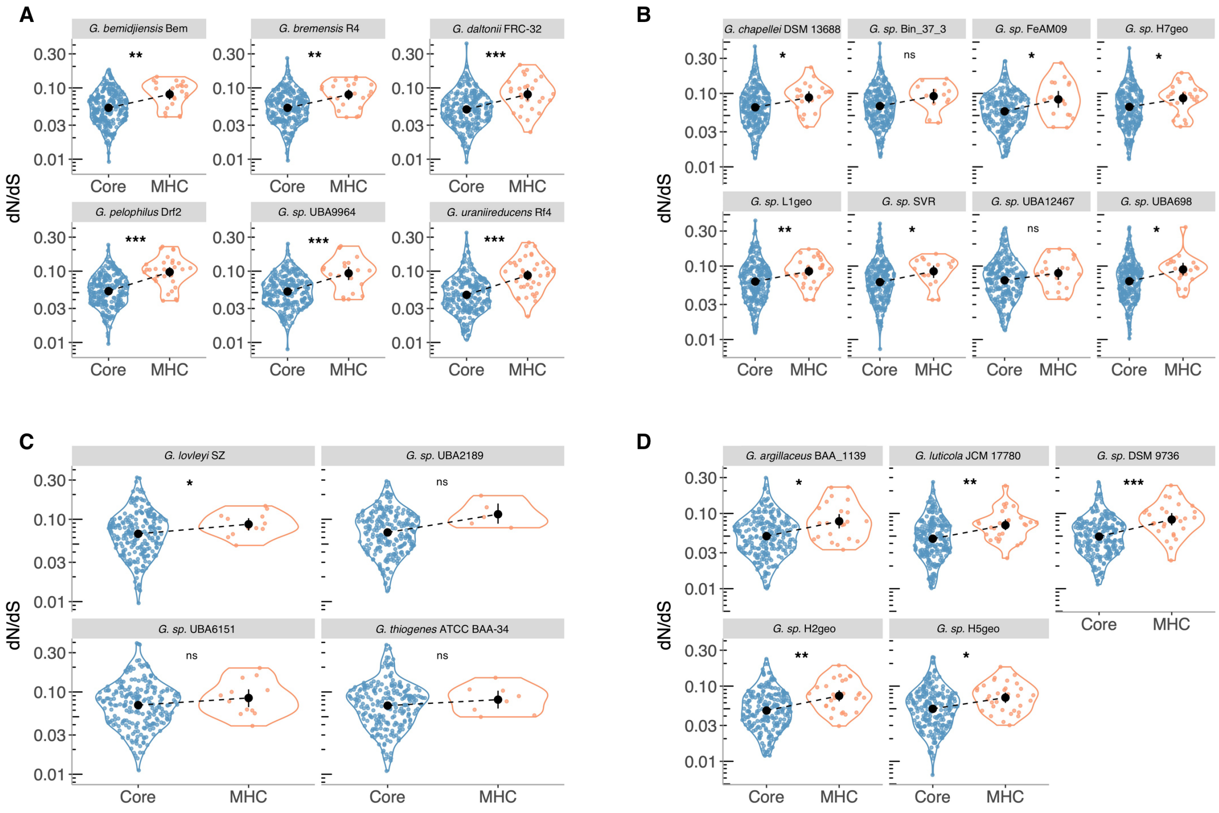

Figure S1. dN/dS analysis of core and MHC proteins in 23 *Geobacter* species, grouped phylogenetically. A) *G. bemidjiensis* Bem clade, B) *G. chapellei* DSM 13688 clade, C) *G. lovleyi* SZ clade, and D) *G. argillaceus* BAA_1139 clade. Significance: * p < 0.05, ** p < 0.01, *** p < 0.001, ns – not significant. *G. sp.* DSM 2909 and *G. sp.* GAC1 had too few MHCs and were omitted.

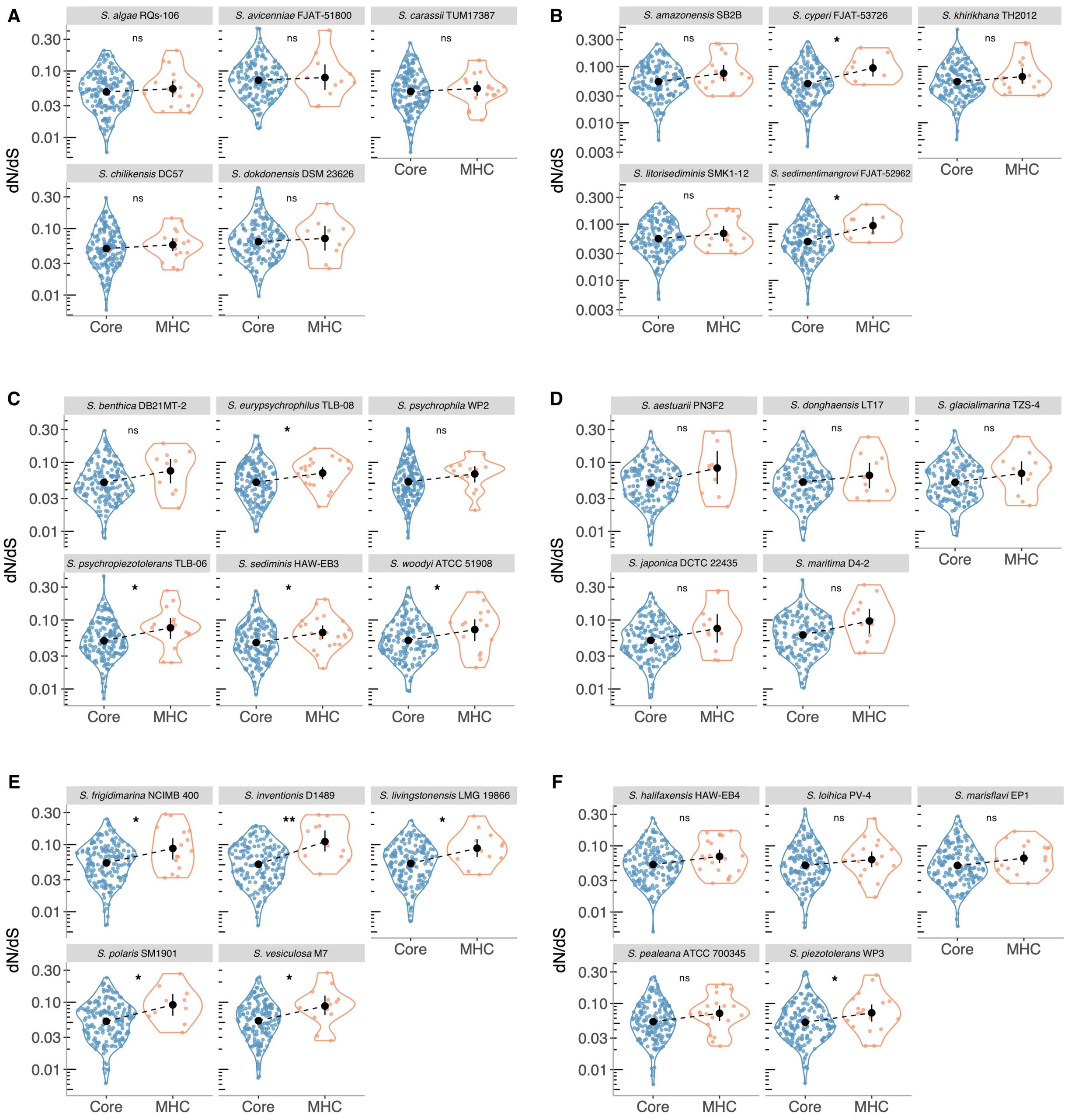

Figure S2. dN/dS analysis of core and MHC proteins in 31 *Shewanella* species, grouped phylogenetically. A) *S. algae* RQs-106 clade, B) *S. amazonensis* SB2B clade, C) *S. benthica* DB21MT-2 clade, D) *S. aestuarii* PN3F2 clade, E) *S. frigidimarina* NCIMB 400 clade, and F) *S. halifaxensis* HAW-EB4 clade. Significance: * $p < 0.05$, ** $p < 0.01$, ns – not significant

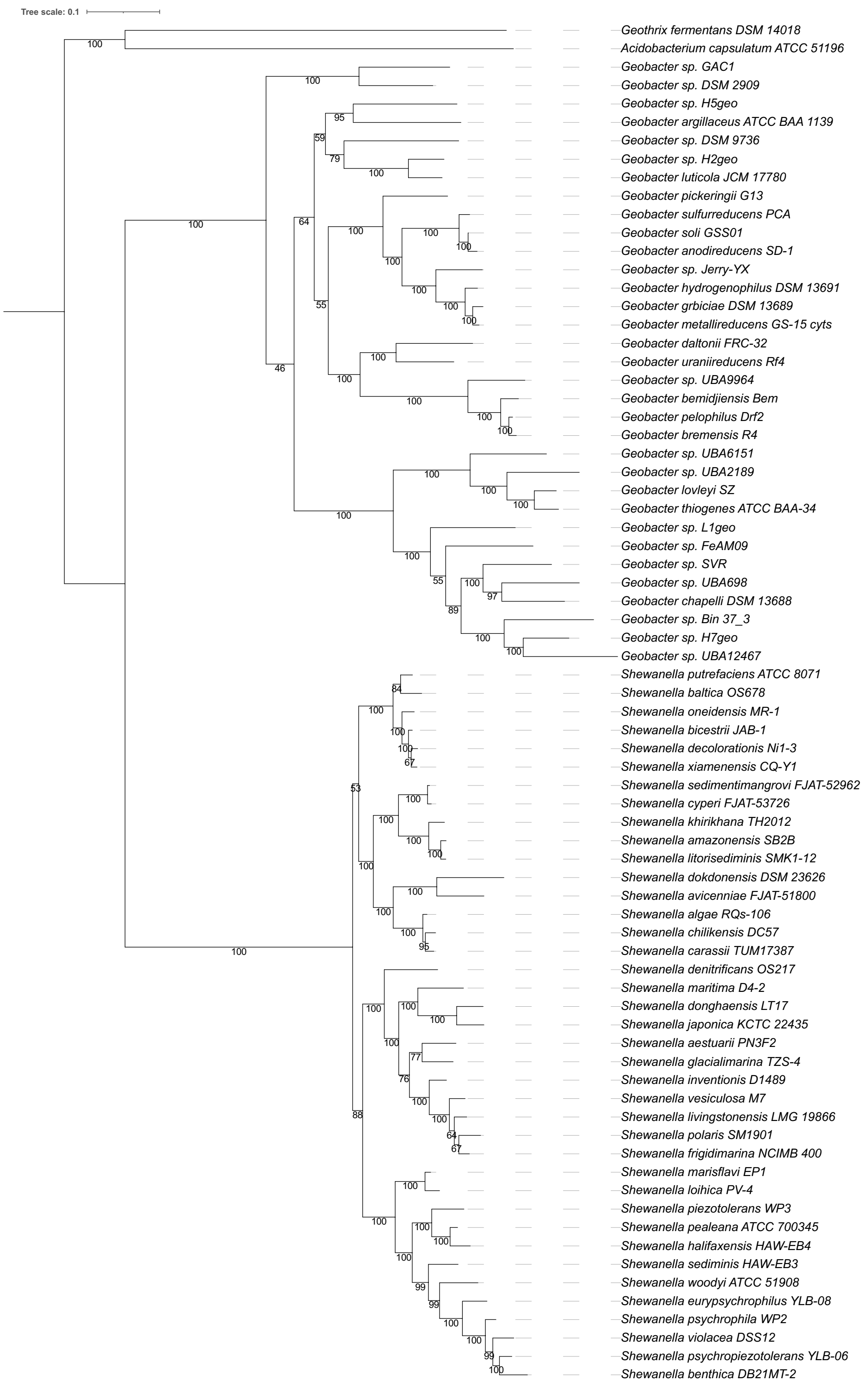

Figure S3. Combined *Geobacter* and *Shewanella* phylogenetic tree with bootstraps.

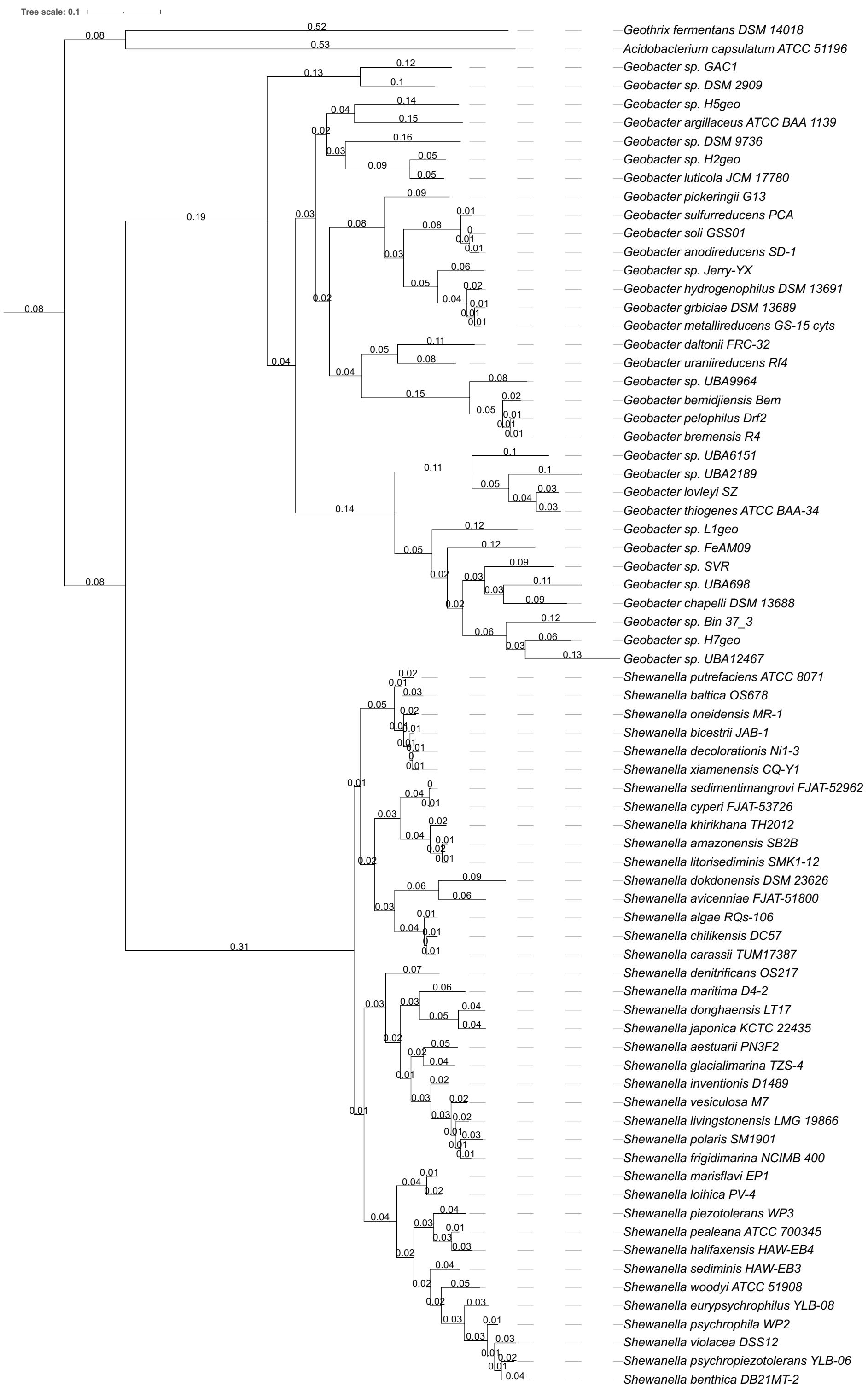

Figure S4. Combined *Geobacter* and *Shewanella* phylogenetic tree with branchlengths. Branchlength data was used to determine genome order and the x-axis scale in Fig. 9.
